# Can we teach a fish how to eat? The impact of bottom and surface feeding on survival and growth of hatchery-reared sea trout parr (*Salmo trutta trutta* L.) in the wild

**DOI:** 10.1371/journal.pone.0222182

**Published:** 2019-09-06

**Authors:** Tomasz Krepski, Robert Czerniawski

**Affiliations:** Department of Hydrobiology, Institute of Biology, University of Szczecin, Szczecin, Poland; Swansea University, UNITED KINGDOM

## Abstract

In this study we attempted to determine the effect of various feeding methods (bottom and surface feeding) used in the hatchery, on the survival and growth rates of hatchery-reared sea trout (*Salmo trutta trutta* L.) in the wild. Rearing was performed in two variants: a bottom-fed group (BFG) and a surface-fed group (SFG). At the end of the rearing time, we observed that BFG fish gathered at the bottom of tank, as opposed to SFG fish, which swam in the whole water column. After 4 weeks of rearing, the fish were released into two similar streams. After about 2 months the fish were captured and the foodbase of the streams were examined. 30 fish from each group have been randomly selected for stomach contents analysis. In the shallow stream the growth rates were better for the BFG fish than the SFG and also a significantly higher number of typical benthic taxa was found in stomachs of the BFG fish than the SFG fish. In the deeper stream more food was found in the stomachs of the SFG fish than in the BFG fish. The analyzed results showed that factors such as stream depth, current velocity, and turbulence can also affect the rearing success of juvenile salmonids in hatchery streams. Bottom feeding fish during rearing has a positive impact only on the fish in shallow watercourses, where there is no turbulence, and the food is not carried by the current drift or washed out from the bottom into the drift.

## Introduction

Anadromous salmonids are one of the most endangered fish in the Baltic catchment area [1, 2, 3]. The main reasons are difficulty in migrating to spawning sites, the harvesting of migrated adult fish, pollution, and the increase in nutrient content in streams, which contributes to the disappearance of spawning places [2, 4]; consequently, juvenile anadromous salmonids are absent in upper parts of streams. Common way of improving the population is to stock streams with hatchery-reared parr or smolt stages of the salmonids [5, 6]; nevertheless, this method is far from perfect because of the high mortality rate of hatchery-reared fry in the wild. Certain researchers believe that the high mortality rate of hatchery-reared fry is because of their lack of necessary foraging skills for survival in the wild [7, 8, 9]. In streams, hatchery-reared fish have difficulties searching for live food; they do not avoid predators, and do not make use of hiding places because they are not familiar with such conditions in the hatchery [10]. Therefore, researchers are looking for methods that allow fish reared in artificial hatchery conditions to develop foraging skills, which thus improves their survivability once they are released into streams [11, 12].

During rearing, such fish are fed with live food or made to avoid predators living in the hatchery streams [7]. Thus, hatchery-reared fish exposed to such conditions can show better survival and growth rates in the wild compared to fish reared on pellet food [9, 13]. Many researchers have reported that an important factor that may increase the survival of fish in the wild is rearing them on live prey or diets supplemented with live food. Furthermore, the process of learning to identify prey is of key significance for the future survival of the fish in the wild [7, 9]. Whilst rearing methods cannot substitute foraging skills learned in a wild environment [10, 14], training hatchery-reared stock beneficial foraging techniques may improve post-release survival.

It is common practice to release fry into smaller hatchery streams, as this is believed to help acclimatise fry to natural conditions. [15]. Subsequently, the fish can be caught and transferred to the target river, from which they migrate to the sea; however, hatchery spawned and reared fish need to stay in the hatchery for several weeks or even months before they are moved to such streams [16, 17]; thus, as described in the above-mentioned cases, the mortality rate is high. We note that the survival and growth rates are different in similar small hatchery streams [9, 13, 18]. This might be because of the difference in the food base and hydrological conditions of the watercourse, which can make it hard for the fry to identify and eat food. Juvenile salmonids feed mostly on insect larvae carried by the stream drift [19, 20]; therefore, we would expect that in streams that are under 20 cm and the velocity does not create turbulence, it is difficult to identify macroinvertebrates in the drift food because these bottom-dwelling organisms are not washed out from the bottom into the drift. Thus, it is worthwhile to try and develop bottom-feeding skills in hatchery-reared fry before they are released into these shallow streams. Indeed, such an attempt is contrary to that followed by nature because the species naturally hunt prey in the drift zone rather than at the surface or at the bottom. Therefore, an important question to address is whether developing artificial feeding skills improves hatchery survival? The bottom-feeding skills can help maintain the salmonids population in shallow, turbulent streams where the food base is rather at the bottom and not in the drift. Because of the decreasing number of streams suitable for salmonids, we should try to allow them to survive in streams that do not have a turbulent current. Sánchez-Hernández and Cobo [21] believe that feeding methods (benthic or surface-feeding) adapted by fish living in a stream may not lead to dietary specialization; however, other researchers think otherwise [22, 23, 24]. To confirm this hypothesis, we performed a laboratory experiment in which we developed a dietary specialization in the fry. Unlike fishes that live in the wild, our hatchery-reared fish were exposed to only one food group, which according to the hypothesis, should lead to specific dietary specializations. Once a dietary specialization was developed in the laboratory, we transferred the fry to a natural watercourse to verify how quickly the fish adapted to the feeding method. Therefore, in this study, we attempted to determine the effect of various feeding methods (bottom- and surface-feeding) used in the hatchery on the survival and growth rates of hatchery-reared sea trout that will be released into a shallow stream. We assumed a hypothesis that the fish feeding on bottom food while rearing would have a better survival opportunity and a faster growth rate in the wild compared to surface-fed fish; consequently, restocking with fry reared on bottom food could improve or help re-establish trout population in the wild.

## Methods

For the experiment, hatchery-reared sea trout fry (*Salmo trutta* L.) was used. The fish came from the Polish Angling Association, hatchery Goleniów, Poland. The larvae were reared for four weeks in six tanks (three replicates for each variant) before releasing them into the wild on April 3, 2017. The volume of water in each tank was 200 L, and the water temperature was maintained between 8 °C (at the beginning of rearing) and 12 °C (at the end of rearing) with a cooling device. Each week, the temperature in the stream that was used for stocking was checked for any changes; these changes were mirrored in the hatchery later. Note that the density per tank was 250 fish with rearing performed in a closed recirculation system in two variants with three replications: a bottom-fed group (BFG) and a surface-fed group (SFG). Both groups were fed on frozen zooplankton (adult cyclopoids and daphnids and Chironomidae larvae). The culture of zooplankton and Chironomidae larvae provided the natural food. For the first two weeks, the food was copepods and adult *Cyclops* sp. For the next few weeks, the food was the same *Cyclops* sp. with the addition of adult daphnids and Chironomidae larvae. Note that the food was defrosted before it was fed to the fish.

The food for the BFG and the SFG was given *ad libitum* and was distributed to the BFG using a 50-mm-diameter tube. The tube inlet to insert the food was placed above the water surface, whereas the tube outlet touched the bottom of the tank. The food fell to the bottom when inside the tube; therefore, the fish did not eat the food from the water column. After spreading out the food at the bottom, the tube was removed; however, the food for the SFG was distributed on the water surface. While the BFG fish ate from the bottom, the SFG fish ate the food from the water column and the surface. Such type of food provisioning was implemented from the start of the experiment. At the end of the rearing time, we observed that the BFG fish gathered at the bottom of tank unlike the SFG fish, which swam in the whole water column. Thus, after observing the distribution of both group fish (BFG and SFG) in the tank, we assumed that habituation to different water-depths had developed. To distinguish between these two groups, the adipose fin of the BFG fry was removed in the third week of rearing; however, the SFG fry were left unmarked. To avoid the influence of the anesthetic drug on fish behavior, both group were treated by the anesthetic drug. Note that all fish survived this treatment and no undesirable behavior was observed. On May 3, 2017, after four weeks of rearing, the fish were released into two similar streams: the Chojnówka and the Trawna. Sampling and experimental activities were performed at locations where specific permission is not required. The streams are ca. 5 km long and flow toward the Odra River near Szczecin, NW Poland. The streambeds are predominantly covered with sand and width of the streams varies from 0.5 to 2.0 m. In summer, the water temperature does not exceed 20 °C; it was 12 °C on the day of the release. On the stocking day, the water current, width, depth, and discharge were 0.27 m s^-1^, 1.20 m, 0.10 m, and 0.03 m^3^ s^-1^ in the Chojnówka and 0.34 m s^-1^, 0.9 m, 0.25 m, and 0.08 m^3^ s^-1^ in the Trawna, respectively.

Note that the distance between the hatchery and the stocking place was 10 km; moreover, we required 15 min to transport the fish, which were in plastic bags filled with oxygen saturated water and none of the fish died during the transfer. To perform the experiment, we sectioned off a 200 m long and 0.7–1.5 m wide area in each stream. Then, in every section (n = 1 per stream), a 5.0 mm size mesh net was extended from the bottom of the stream to 1 m above the water surface. We released a total of 400 fish into every section, namely, 200 fish from each of the two feeding groups. Note that the nets were cleaned every day.

On August 3, 2017, the fish were captured with an electric fishing gear (Hans Grassl ELT60 II, Germany) along the entire length of every section in each stream. On that day, the water temperature was 19 °C. To ensure that all the fish were caught, the procedure was performed three times on the same day by three people: two people collected the fish and the third person walked 50 m behind to check that none of the stunned fish were carried down the river. There were no other predatory species as all other predatory fish had already been captured using an electric fishing gear before the stream was stocked with the hatchery-reared trout. Although mammalian and avian predators are present in the catchment area they were not observed in high numbers during the study and were therefore assumed to have low impact on trout survival.

On the day of the capture, the macroinvertebrates used as the fry food base were collected from the experimental sections of each stream. The samples were collected along the entire profile of the riverbed with a rectangular scraper (0.20 m) and across a width of 1 m; they were then transported to the laboratory and identified. All the captured fish were measured, weighed, and humanely euthanized in the laboratory after an overdose of MS-222, and then their stomach content was checked. From each group, 30 fish were randomly selected for stomach contents analysis, and the organisms found were identified to the lowest taxonomic level possible. All terrestrial organisms were categorized as non-aquatic organisms. All captured fish were measured, weighed, and killed in the laboratory with an overdose of MS-222 to verify their stomach contents. All treatments were implemented in accordance with Polish law.

The condition factor (K) was calculated using the following formula: K = 10^5^ ML^-3^, where L is the fork length (mm) and M is the mass (g). Statistical significance of the differences in the number of invertebrate taxa in the fish stomachs between the BFG and the SFG was determined using the Mann–Whitney U test for comparing two independent groups (P < 0.05). The significant differences between the BFG and the SFG in fork length, mass, and the condition factor of the fish captured from streams were determined using one-way variance analysis (one-way ANOVA) and the *post-hoc* Tukey’s test (P < 0.05). Moreover, significant differences between the BFG and the SFG in fork length, mass, and the condition factor of the fish after rearing were determined using the Mann–Whitney U test for comparing two independent groups (P < 0.05).

Differences in the stomach content of fishes, based on the number of macroinvertebrate individuals in fish stomachs between the BFG and the SFG, and between the streams (Chojnówka and Trawna) were tested with PERMANOVA with Bray–Curtis distances (10000 replicates) using the Vegan 2.4 package for R. The dependent variable was the amount of organisms eaten by the fish and the grouping was fish (BFG and SFG) and stream (Chojnówka and Trawna).

## Results

After rearing, both the fish groups demonstrated similar survival rates: BFG was 96% and SFG was 98%. The SFG achieved significantly higher values of fork length and mass than the BFG (Mann–Whitney U test for fork length: df = 1, P < 0.0016; and Mann–Whitney U test for mass: df = 1, P < 0.0018) (Table 1).

**Table 1:** Final results of rearing and basic characteristics (mean ± SD) of sea trout fry in the stocking experiment.

Group	n	Fork length (mm)	Mass (g)	Condition factor
BFG	30	29.35 ±1.11	0.227 ± 0.025	0.90 ± 0.09
SFG	30	31.80 ±1.97	0.293 ± 0.055	0.91 ± 0.08
*P* value		0.0016	0.0018	0.8438

BFG, bottom-fed group; SFG, surface- fed group

After being captured from the streams, the mean survival rate of the fish from the shallower Chojnówka was 25% for the BFG and 19.5% for the SFG; however, that of the fish from the deeper Trawna was 15.5% for the BFG and 21.5% for the SFG. The mean values of fork length and mass of the BFG from the Chojnówka were significantly higher than those of the SFG (ANOVA for fork length: F = 23.06, df = 1, P < 0.0001; Tukey’s test: P < 0.0001 and ANOVA for mass: F = 17.67, df = 1, P < 0.0001, Tukey’s test: P < 0.0001) (Table 2). Despite greater fork lengths and mass values in the BFG than in the SFG from the Trawna, the differences between the groups were not significant (P > 0.05). The differences between values of the condition factor in the BFG and the SFG in both streams were also insignificant (P > 0.05); however, in the Chojnówka, this parameter was clearly higher for the BFG than for the SFG (Table 2).

**Table 2:** Mean values ± SD of growth parameters of sea trout in the shallower Chojnówka and deeper Trawna streams.

Stream	Group	n	Fork length (mm)	Mass (g)	Condition factor
Chojnówka	BFG	45	86.97 ±10.41	6.94 ± 1.83	1.06 ± 0.19
	SFG	39	77.71 ± 6.49	5.47 ± 1.28	1.15 ± 0.12
	*P* value		<0.0001	0.0001	0.0154
Trawna	BFG	30	103.08 ±17.00	12.59 ± 5.25	1.09 ± 0.15
	SFG	44	101.31 ± 9.69	11.54 ± 3.09	1.11 ± 0.15
	*P* value		0.4058	0.3966	0.3874

BFG, bottom-fed group; SFG, surface- fed group

### Food base

For both streams, the food base was mostly composed of gammarids and chironomids; moreover, in Trawna and, to a lesser extent, in Chojnówka, high abundance of Diptera larvae was noted. In both these streams, similar number of taxa noted; however, in Trawna, the organisms were more abundant (Table 3).

**Table 3:** Mean density of macrozoobenthos as a food base on capture day in two examined streams. (ind. m^-2^).

Taxa	Chojnówka	Trawna
Sphaeriidae	156	232
Oligochaeta	-	64
Erpobdellidae	4	-
*Asellus aquaticus*	28	-
*Gammarus* sp.	348	1876
Nemouridae larva	4	4
Baetidae larva	68	12
Ephemeridae larva	-	52
Mesoveliidae	4	-
Vellidae	8	-
Limnephilidae larva	12	12
Haliplidae larva	-	28
Chironomidae larva	276	368
Ceratopogoniidae larva	-	4
Dixidae larva	-	4
Limoniidae larva	44	20
Ptychopteridae larva	4	180
Simuliidae larva	-	100
Tabaniidae larva	12	68
Hydracarina	8	-

### Stomach content

**Fig 1:**
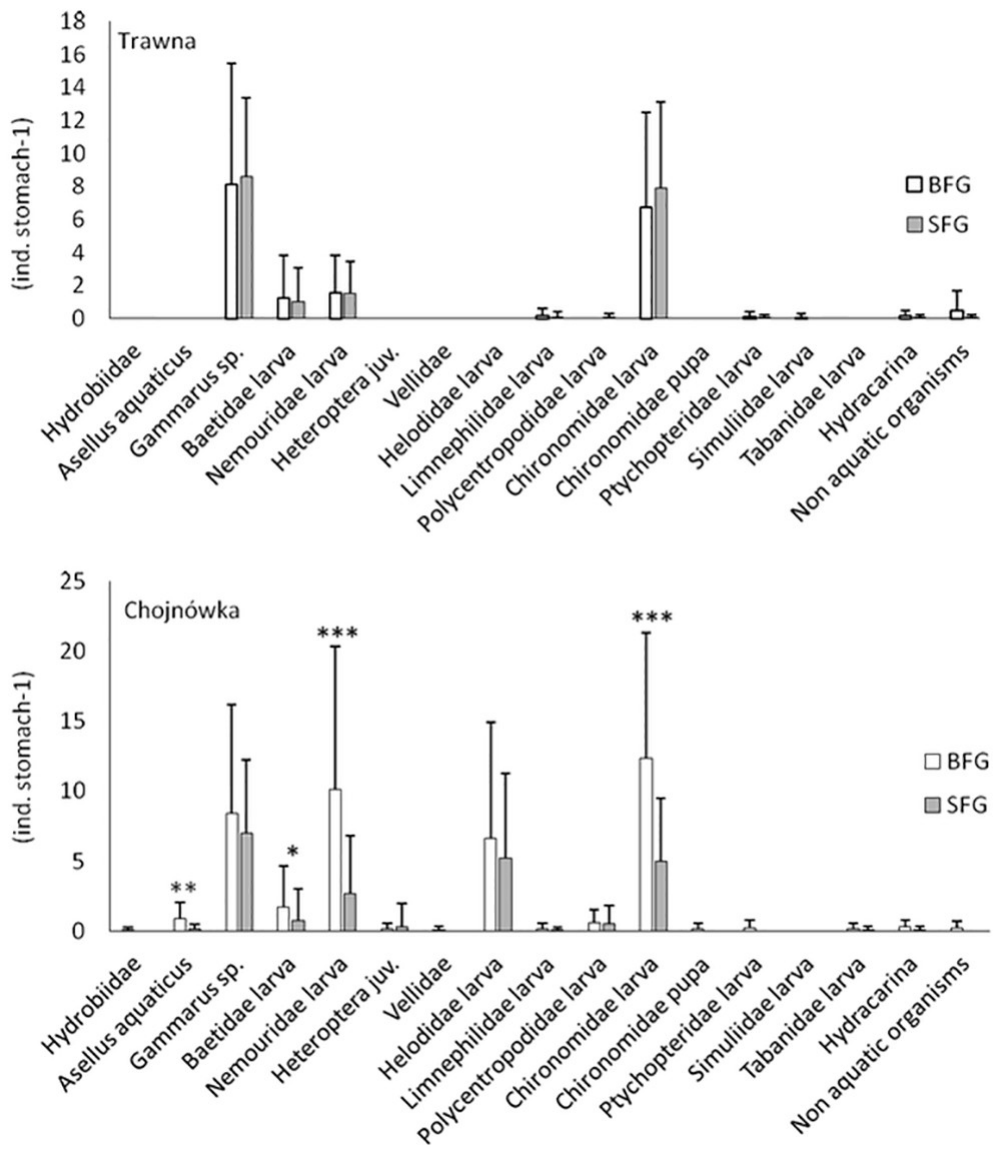
Mean density of macrozoobenthos individuals in stomachs of hatchery-reared trout in streams used for stocking. BFG–Bottom Feeding Group, SFG–Surface Feeding Group. Asterisk = significant differences between BFG and SFG in number of taxa in stomach content; *P < 0.05, **P < 0.01, ***P < 0.001.

The most common taxa in the stomachs of fish from both streams were gammarids and larvae of chironomids (Fig 1). Apart from these taxa, Neumoridae and Helodidae larvae were predominant in the stomachs of fish from the Chojnówka. There were a higher number of almost all organisms in the stomachs of the BFG than the SFG captured from the Chojnówka (Fig 1). In this stream, the number of *Asellus aquaticus*, Baetidae larvae, Nemouridae larvae, and Chironomidae larvae were significantly higher in the stomachs of the BFG than the SFG fish (P < 0.05) (Mann–Whitney U test for *Asellus aquaticus*: df = 1, P < 0.0002; for Baetidae larvae df = 1, P < 0.0333; for Nemouridae larvae df = 1, P < 0.0005; for Chironomidae larvae df = 1, P < 0.0007) (Fig 1). In the BFG and the SFG fish stomachs, the number of each observed taxa was similar (P > 0.05). Note that, in the Trawna, the food base was three times more abundant than in the Chojnówka (Table 3). A PERMANOVA indicated there were significant differences in number of organisms in fish stomachs between these fish groups (P < 0.001). This analysis showed also significant differences in stomach content between streams (P < 0.001); moreover, there was also significant interaction between fish group and stream (P < 0.001).

## Discussion

### Growth of the fish during the rearing period

A higher survival rate of the SFG during rearing may be because of the long time it takes for the food to drift and fall from the surface to the bottom. The SFG fish had more time to eat the food from the water column compared to the BFG. Several researchers have reported that salmonid fry mostly feed on food drifting with the current than the food found at the bottom of the stream [19, 20]. As observed in our previous experiment, fish reared on live drifting food, which stays for a long time in the water column, achieve faster growth rates than fish reared on a dry pellet-based diet that stays in the water column for a brief period [9, 13]. This pattern was observed in the first 2–3 weeks of rearing. We were aware that rearing on surface food can give better results for fish growth compared to bottom food. However, we assumed that the surface food, which is less available to drift-fed fish, was beneficial for developing the foraging skills of the salmonid fry, which was later stocked into the shallow hatchery streams. Furthermore, at the end of the laboratory rearing period, we observed strong feeding behaviors in fish. In particular, the SFG fish mainly fed close to the water surface, whereas the BFG fish ate from the bottom.

### Growth of the fish in the wild

Many researchers have indicated that rearing on a natural diet, such as live food, rather than an artificial one is an essential factor that may increase fish survival in the wild [9, 11, 12, 25, 26]. Therefore, feeding methods may significantly impact the survival of hatchery-reared fish released into the wild. Based on these results, we expected that feeding methods impact fish feeding behaviors in a stream. This relationship can be clearly observed in the shallow waters of Chojnówka, for which the growth rates were better for the BFG fish than the SFG. Moreover, a significantly higher number of typical benthic taxa was found in stomachs of the BFG fish than the SFG fish from the Chojnówka. This suggests that feeding methods employed in the hatchery tanks positively influence feeding behaviors in the wild. Therefore, in the case of the Chojnówka, it is reasonable to assume that, during the rearing period, the fish had habituated to different water-depths that influenced their survival rate and growth in natural conditions. According to Paszkowski and Olla [25] and Brown and Laland [7], the process of learning to identify prey is of key significance to the future survival of the fish in the wild.

When comparing the growth rates of fish from the Chojnówka and the Trawna, we must consider varying environmental conditions and hydrological conditions in particular of both streams. The Chojnówka is shallower, its current velocity is rather low, and it exhibits weak turbulence conditions; therefore, benthic organisms are washed out to a small extent from the bottom into the drift. Hence, the food is more likely to be found at the bottom; therefore, the BFG fish may have habituated to bottom-feeding during the rearing stage, which in turn resulted in better growth rates in the wild.

Unlike the Chojnówka, the Trawna is deeper and is characterized by greater current velocities and turbulence; consequently, the food was washed out of the bottom into the drift more. In fact, the fish fed in the drift because they found food there; thus, in many cases, more food was found in the stomachs of the SFG fish from the Trawna than in the BFG fish. Furthermore, the SFG fish that had learned to search for food drifting in the water column also ate the same invertebrates in the stream. As mentioned previously, the Trawna was richer in food base than the Chojnówka; therefore, more favorable food conditions and availability for drift-fed salmonids in the Trawna ensured better biometrics rate of the SFG fish compared to the Chojnówka. Moreover, in food-rich hatchery streams, where the food is carried by the current drift, the feeding method during the rearing is unlikely to have a significant impact on the fish growth rate. In such conditions, juvenile salmonids opt for natural feeding behaviors, i.e., eating the food carried by the current drift.

However, this was not observed with the Chojnówka. The stream conditions, such as shallow depth and lack of turbulence, benefitted the fish which searched for food at the bottom. Hence, in this case, the rearing could have influenced the fish feeding behavior and growth rate. Nonetheless, the fish could have been motivated to search for food at the bottom because of the environmental conditions, namely, the lack of food in the drift. We believe it would be worth examining this phenomenon for several environmentally similar streams. Furthermore, it could be assumed that the shallower the watercourse of the hatchery stream, the higher is the impact of the feeding method on the stocking material during the rearing. However, an important question needs to be addressed: how long will the acquired behavior persist in the fish and what happened with the BFG after reaching the sea?

### Food of the fish in the wild

Our results can be compared to observations by Orlov et al. [27] who studied feeding behaviors of cultured and wild Atlantic salmon juveniles. They found that cultured salmon primarily fed on the bottom of the water column at slower current velocities, whereas wild fish primarily fed in the drift of the water column at faster current speeds. Moreover, the content of drift items in the wild parr diet was found to be 47% higher compared to that of the cultured parr. Based on these results, we can hypothesize that the SFG habituated to a higher water level, therefore ate drift food in tanks, and were later able to effectively catch prey in the deeper Trawna stream, unlike the BFG which habituated to deeper water levels and consequently ate benthic prey.

Note that the fork length, mass, and condition factor values are similar to those reported for other salmonid species of the same age [18, 20, 28, 29]. High similarity in the fish growth rate and no significant differences in condition factor values between the two streams demonstrate that both streams offer food and environmental conditions that are sufficient for fry growth at a certain fry density. It would be worth analyzing the taxonomic composition of the stomach content of fish from both these experimental groups. In the aforementioned experiment, the Chironomidae larvae were predominantly seen in the stomachs of trout parr reared primarily on the Chironomidae larvae [13]. Moreover, these organisms were frequently a common component of the salmonid parr food base in the wild [18, 19, 30]. Note that the BFG fish ate more taxa in the wild. Perhaps, the fish were more likely to search for food at the bottom of the stream because they got used to this process in the tanks. In fact, more benthic organism taxa can be found at the stream bottoms than in the drift.

Oftentimes, salmonids eat Chironomidae larvae and crustaceans in relatively high abundance [18, 19, 28]. In this study, a reasonably high number of these taxa was observed in the stomachs of the two groups. Nevertheless, a significantly higher number of taxa was found in the stomachs of the BFG fish from the Chojnówka than the SFG fish. Note that the number of crustaceans was relatively high in each stream and the fish could have eaten this readily available food. Therefore, the established pattern was not surprising. Similar to the case of the Chironomidae, the fish might simply prefer this component of the food base. Nevertheless, the BFG in the Chojnówka might be better at capturing Chironomidae larvae and crustaceans in the wild than the SFG. Although various feeding methods have been used in the present experiment and in the past studies [9, 13], we noticed similar survival rates of fish in the wild.

Furthermore, the analysis of the contribution of food base components has shown that the BFG fish in the Chojnówka preferred typical benthos taxa. Moreover, other fish from the Chojnówka and the Trawna primarily preferred taxa of invertebrates that could also be carried by the current drift. As mentioned above, this difference is a consequence of different morphological conditions in the two streams. Juvenile salmonids prefer to eat food from the water column and surface rather than the bottom of the watercourse [19, 31, 32]; however, our results do not support this claim. This is especially true in the case of the BFG fish from the Chojnówka because the predominant food component found in their stomachs were typical benthic organisms. The Chojnówka is a shallow river; therefore, the fish are more likely to reach the bottom and encounter benthic organisms rather than trying to capture themin the water column. However, the opposite was observed in the Trawna, which is a deeper stream. Stradmeyer and Thorpe [19] reported that juvenile salmonids mostly feed on organisms carried by the current drift and eat benthic species less frequently.

## Conclusion

To summarize, the present study demonstrated that the habituation of trout to various water-depths, achieved by implementing different feeding methods in hatcheries, can increase post-release growth and survival in hatchery streams of the appropriate habituated depth. Stocking success also depends on the morphological, physicochemical, and food conditions in a watercourse. Factors such as stream depth, current velocity, and turbulence affect the rearing success of juvenile salmonids in hatchery streams: the deeper the watercourse and greater the turbulence, the better are the feeding conditions for the juvenile salmonids.

Therefore, bottom-feeding during rearing has a positive impact only on the fish in shallow watercourses, where there is no turbulence and the food is not carried by the current drift or washed out from the bottom into the drift. Moreover, shallow streams should be used as hatchery streams if the stocking material has been appropriately prepared beforehand. We believe future studies can pointed the hydrological conditions that a hatchery stream should have to provide easy access to food.

## Supporting information

S1 File Canweteach_rawdata.xls. Raw data obtained during the study period.
(XLS)
